# Harmony in chaos: understanding cancer through the lenses of developmental biology

**DOI:** 10.1002/1878-0261.13594

**Published:** 2024-01-29

**Authors:** Jonas Van Lent, Arianna Baggiolini

**Affiliations:** ^1^ Institute of Oncology Research (IOR) Bellinzona Institutes of Science (BIOS+) Switzerland; ^2^ Faculty of Biomedical Sciences Università della Svizzera Italiana Lugano Switzerland

**Keywords:** embryonic development, hPSC‐based cancer models, melanoma, pluripotent stem cells

## Abstract

When we think about cancer, the link to development might not immediately spring to mind. Yet, many foundational concepts in cancer biology trace their roots back to developmental processes. Several defining traits of cancer were indeed initially observed and studied within developing embryos. As our comprehension of embryonic mechanisms deepens, it not only illuminates how and why cancer cells hijack these processes but also spearheads the emergence of innovative technologies for modeling and comprehending tumor biology. Among these technologies are stem cell‐based models, made feasible through our grasp of fundamental mechanisms related to embryonic development. The intersection between cancer and stem cell research is evolving into a tangible synergy that extends beyond the concepts of cancer stem cells and cell‐of‐origin, offering novel tools to unravel the mechanisms of cancer initiation and progression.

## Graphical abstract

As our comprehension of embryonic development expands, it unveils insights into how certain mechanisms exploited by cancer cells operate and why they are hijacked. Developmental biology has also paved the way for crucial advancements in technologies based on human pluripotent stem cells (hPSC), offering instrumental tools for understanding and tackling various human diseases, including cancer.

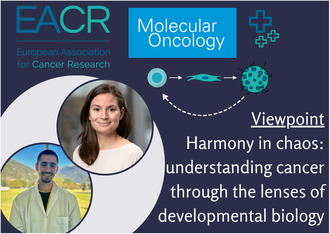

**Abbreviations**
hESC: human embryonic stem cells
hPSC: human pluripotent stem cells
iPSC: induced pluripotent stem cells
NC: neural crest

## 1 Developmental biology sheds light on cancer mechanisms

Many core concepts in cancer biology have been initially identified and studied by developmental biologists. Among some of those fundamental concepts, we find the epithelial–mesenchymal transformation (EMT), characterized in the 1960s by Elisabeth Hay, who observed cells migrating from the primitive streak of an embryo. Trophoblast cells, which are highly proliferative and invasive cells in developing embryos, exemplify how immune tolerance mechanisms must be developed to support healthy human development. These mechanisms are also often co‐opted by cancer cells to evade the immune system and promote their growth. Conrad Hal Waddington's concept of the epigenetic landscape illuminates the diverse pathways progenitor cells traverse during development to become specialized, functional cell types. This model encompasses cellular competence, differentiation, and plasticity—features mirrored in cancer cells during malignant transformation, tumor progression, and resistance to treatment. Because of the similarities between development and cancer, cancer research has been particularly interwoven with developmental biology research. A notable example of this convergence is evident in studies of melanoma.

Melanoma, a cancer of melanocytes, is one of the most aggressive skin cancers because of its high metastatic potential. During development, melanocytes originate from the neural crest (NC), a multipotent progenitor population that gives rise to various cell types, including melanocytes, cells of the peripheral nervous system, and craniofacial skeleton, among others. Insights from developmental biology shed some light on mechanisms exploited by cancer cells during initiation, growth, metastasis, and therapy resistance. Numerous genes associated with the NC are indeed aberrantly expressed or upregulated in melanoma cells. To give a few examples, the neurotrophin receptor CD271 (also called p75NTR and NGFR) is a NC marker also expressed by melanoma cells, and it regulates melanoma phenotype switching and metastatic spreading [1]. SOX10, a transcription factor expressed by migratory NC cells, has been shown to be highly expressed by melanoma cells and required for tumor formation [2], while SOX9, a NC specifier [3], rather regulates tumor invasion [4]. Studies employing zebrafish melanoma models have revealed the dependency of melanoma initiation on NC‐specific genes, such as the zebrafish‐specific *crestin* [5]. Finally, shared metabolic and nucleotide biosynthesis programs between NC and melanoma cells underscore their similarities [6, 7].

Our deepening understanding of developmental processes unravels mechanisms exploited by cancer cells, but also promotes the generation of innovative models, including human pluripotent stem cell (hPSC)‐based technologies, which pave the way for novel insights into cancer development and therapeutic strategies.

## 2 Human pluripotent stem cells

Human PSC have the remarkable ability to develop into almost any cell type of the human body. They are characterized by their capacity for self‐renewal and their potential to differentiate into a vast array of specialized and functional cell types. Human PSC include embryonic stem cells (hESC), derived from the inner cell mass of blastocysts [8], and induced pluripotent stem cells (iPSC) reprogrammed from somatic cells [9, 10]. Human PSC‐based technologies have opened the possibility of investigating human cell types that were previously difficult to access due to ethical or technical constraints. These technologies enable the exploration of human progenitor cells, bypassing ethical limitations associated with accessing human fetal tissues. Similarly, the study of human somatic cells has been streamlined, avoiding the challenges of isolating and culturing cells from *postmortem* tissues or biopsies. Furthermore, iPSC‐based technologies facilitate the investigation of patient‐specific cell types, offering insights into the intricate genetic underpinnings of various diseases.

## 3 The use of hPSC in cancer research

**Fig. 1:**
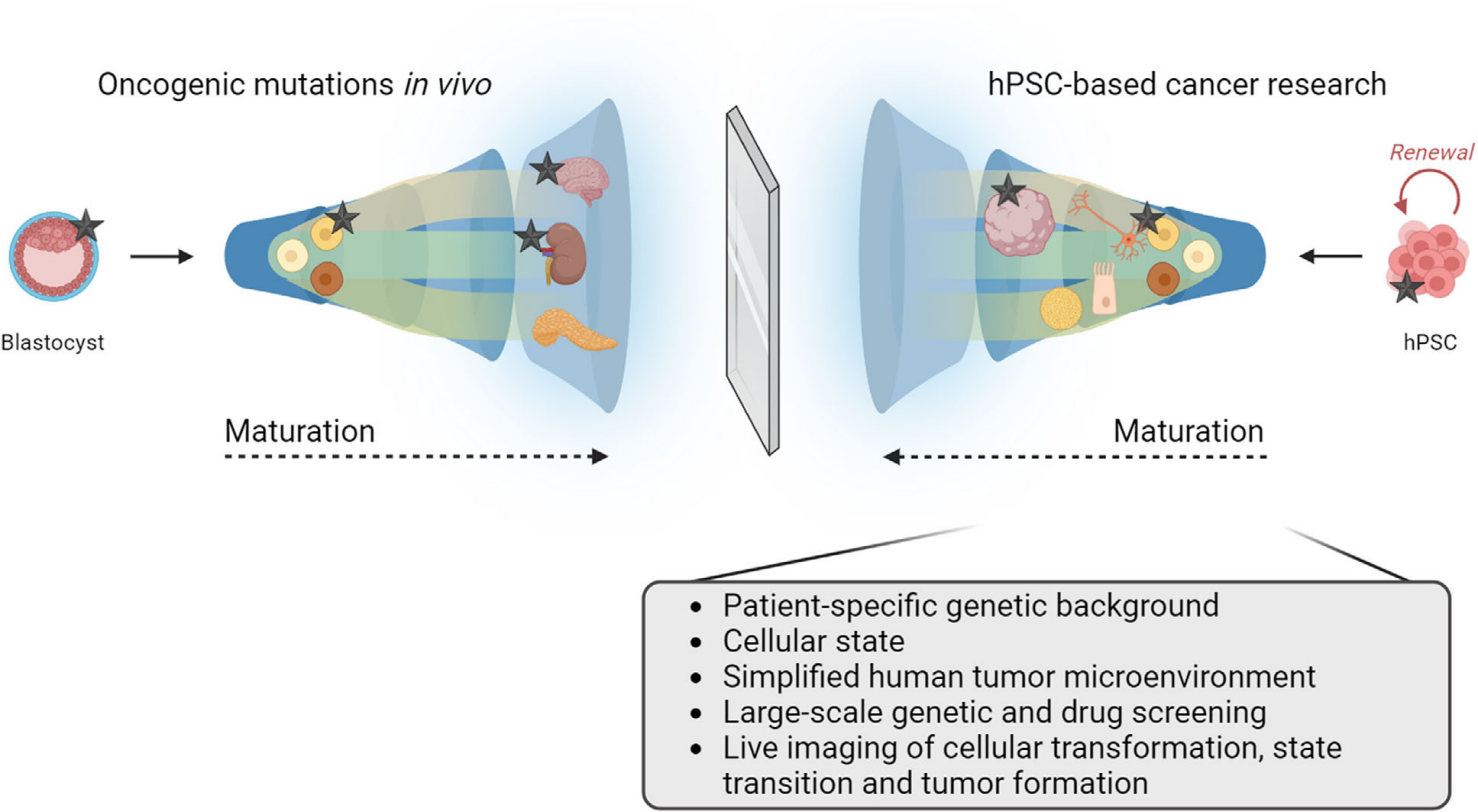
hPSC‐based cancer research. The drawing on the left illustrates the acquisition of oncogenic mutations at various cellular maturation stages: oncogenic mutations occurring as germline mutations, during development, or in adult tissues are denoted by black stars. Conversely, the schematic representation on the right portrays how hPSC‐based technologies allow the recapitulation of tumorigenesis across diverse human cell types at different maturation stages (progenitor cells or fully differentiated cell types). At the center, a metaphorical mirror symbolizes our attempt to reflect cellular maturation, plasticity, and mutation‐induced transformation using hPSC‐based technologies. The figure has been created with Biorender.

While hPSC‐based technologies have seen widespread applications from developmental biology to neurodegeneration research, an increasing body of studies is now leveraging these advancements for cancer modeling (Fig. 1). Studies encompassing glioblastoma [11] and other brain‐related tumors [12], melanoma [13], and ovarian cancer, among others, have used hPSC‐based models to recapitulate the disease in a human context and capture elusive cellular states that are otherwise challenging to replicate and sustain in culture. For instance, an hPSC‐based H3.3G34R‐mutant glioma model has shown that human forebrain progenitors, but not hindbrain progenitors, are oncogenic competent and readily transformed by *H3.3G34R*, *ATRX*, and *TP53* mutations [11]. Human PSC‐derived brain organoids [14] have also been established to model glioblastoma formation and growth [15, 16]. Patient iPSC‐derived fallopian tube organoids carrying BRCA1 mutations have been used to model ovarian cancer and recapitulate human fallopian tube epithelial malignant cell transformation. Of note, the iPSC‐derived organoids that showed the greatest pathology were those derived from patients affected by the most aggressive ovarian cancer, suggesting their potential use in predicting the clinical severity before the onset of the disease and offering a personalized platform for drug screening [17]. Our recent hPSC‐based melanoma model has been instrumental in shedding some light on the role of the cellular state in response to oncogenic mutations [13]. This model has shown that within an identical mutational framework, certain developmental epigenetic factors, such as ATAD2, support the transformation of progenitor cells like NC cells and melanoblasts upon acquisition of oncogenic mutations. The data also showed that mature melanocytes are less oncogenic competent than progenitor cells, requiring additional loss of tumor suppressors or epigenetic reprogramming to undergo malignant transformation. On this line, studies have shown that melanoma cells can be reprogrammed into iPSC and then differentiated into mature cell types, such as fibroblasts, but that those cells lose their tumorigenic potential [18]. This further underscores the pivotal role of cellular states in driving tumorigenesis. These examples are just a glimpse of the applications of hPSC‐based technologies in cancer research. Several other hPSC‐based cancer models exist, such as models involving tumors of NC‐derived lineages beyond melanoma, e.g. neurofibroma [19] and neuroblastoma [20].

Human PSC‐based technologies offer the remarkable opportunity to capture early molecular events in cancer initiation, trace cancer origins, and recapitulate cancer progression, providing crucial insights into understanding early molecular and genetic changes. Cancer research has only started to explore and harness the potential of hPSC‐based technologies, which harbor tremendous promise for future research endeavors.

## 4 The perspective of hPSC‐based technologies in cancer research

In the coming years, we expect that hPSC‐based technologies will drive investigations into several critical areas. Among some of them, we anticipate:

- Oncogenic competence: Understanding why some cells, which share the same lineage trajectory but at different maturation states, show differential responses to oncogenic mutations and the potential for malignant transformation in a human context.
- Cellular state, plasticity, and response to treatment: Investigating hPSC‐derived cells at different maturation states under diverse therapeutic strategies.
- Simplified human tumor microenvironment: Leveraging hPSC‐based technologies to recreate some specific aspects of the tumor microenvironment and delineate crucial mechanisms of cellular interaction and niche remodeling.
- Patient‐specific drug and genetic screens: Utilizing iPSC‐based technologies to generate scalable numbers of patient cell types of interest will facilitate both drug and genetic screening in specific cellular types and maturation states.

It is noteworthy that while hPSC‐based models offer valuable insights, they are not without limitations. For instance, challenges persist in achieving advanced maturation states in hPSC‐derived cell types. However, depending on the scientific inquiries, hPSC‐based models might serve as innovative tools to address longstanding questions within a human‐centric and potentially patient‐specific context.

## Conflict of interest

The authors declare no conflict of interest.
